# Cognition, Behavior, Sexuality, and Autonomic Responses of Women with Hypothalamic Amenorrhea

**DOI:** 10.3390/brainsci12111448

**Published:** 2022-10-26

**Authors:** Carlo Pruneti, Sara Guidotti

**Affiliations:** Clinical Psychology, Clinical Psychophysiology and Clinical Neuropsychology Laboratory, Department of Medicine and Surgery, University of Parma, 43126 Parma, Italy

**Keywords:** hypothalamic amenorrhea, stress, reproduction, sexuality, mental health

## Abstract

(1) Background: Functional Hypothalamic Amenorrhea (FHA) can be caused by the hyper activation of neuro-endocrine responses to stress. Among other endocrine factors and hypothalamic dysfunctions, the psychophysiological stress response can very frequently lead to an inhibition of the gonadal–pituitary axis. The aim of this study was to investigate the level of neurovegetative activation in a group of young women affected by this condition. (2) Methods: Twenty-five women (mean age = 21.1 ± 4.34) with FHA were consecutively recruited. Information on psycho-physiological distress was collected through a Psychopathological assessment (with the administration of three psychometric tests) and the Psychophysiological Stress Profile (PSP). Their data were compared with a control group. (3) Results: In the PSP, the patients displayed significantly higher values compared to controls in terms of the parameters of muscle tension (sEMG), skin conductance (SCL/SCR), heart rate (HR), and peripheral temperature (PT). Furthermore, autonomic hyper-activation at rest, marked reactivity to stress, and reduced recovery were seen. Moreover, a condition characterized by psychological distress (anxiety and somatic complaints, depressed and irritable mood, obsessive-compulsive traits) emerged. (4) Conclusions: The results highlight autonomic hyper-activation in FHA, which is also associated with psychological distress. Considering that FHA is a condition that affects multiple systems between mind and body, a multimodal, multidimensional, and multidisciplinary assessment of stress is becoming an emerging need.

## 1. Introduction

Amenorrhea is the medical term for the absence of menstruation in women of childbearing age. This condition can be primary or secondary. Primary amenorrhea corresponds to the non-arrival of the menarche while secondary amenorrhea is defined as the absence of menses for more than three months and/or a menstrual cycle that persistently exceeds 45 days [1,2]. Functional Hypothalamic Amenorrhea (FHA) and Poly-Cystic Ovarian Syndrome (PCOS) are the most common causes of secondary amenorrhea [1,2]. A set of parameters is needed in order to diagnose FHA. More specifically, focused clinical and family history examinations, a negative pregnancy test, and blood tests to assess gonadotrophins as well as thyroid function, prolactin, and Anti-Mullerian Hormone (AMH) levels should be carried out. The assessment of gonadotrophin levels usually reveals Hypogonadotrophic Hypogonadism (HH), with Gonadotropin Releasing Hormone (GnRH) deficiency, resulting in low levels of Luteinizing Hormone (LH), Follicle Stimulating Hormone (FSH), and estradiol [2].

Considering that FHA is a type of secondary amenorrhea without an identifiable structural organic cause, the term “functional” highlights that this condition can be reversed once the physiological cause is identified and managed [2]. The mechanism of FHA relates to a reduction in GnRH pulsatility, which results in a reduced LH pulsatility and circulating LH and FSH levels with a differential decrease in LH relative to FSH levels. The reduced LH pulsatility and low estradiol levels subsequently result in the anovulation and cessation of menstrual periods [1,2,3].

In addition, interpersonal and relational functioning is negatively affected in individuals with FHA, as are their sexual and reproductive lives. In particular, these women complain of different sexual disturbances related to the various phases of the sexual intercourse, such as a decrease in libido, poor sexual arousal, poor lubrication, and consequent symptoms of discomfort and pain during penetration [4]. All of these aspects are associated with the presence of alterations in the self-image: feelings of inferiority and inadequacy are often described by these people, as well as internalizing and/or externalizing symptoms (irritability, fatigue, etc.) [5].

In general, the diagnosis of FHA is based on the exclusion of other causes of non-menstruation, including organic and anatomical factors [1,5,6,7,8]. The main determinant of the disorder is a combination of psychosocial and metabolic stress. More specifically, predisposing factors include low energy availability, nutrient deficiencies, an excess of physical activity, absence of endometrium regeneration, abnormal sleep, emotional tension, unmanageable chronic or severe stress, and dysfunctional behavior [1,5,8,9].

The onset of amenorrhea is relatively common in the psychological setting because distress is a key part of the clinical history that may include many potentially stressful events (i.e., changing schools, relationship breakdowns, and family bereavement). In fact, in some cases, psycho-social stressors correspond to a perception of threat to the homeostasis of the individual which then reacts with a stress response. Therefore, during the “fight or flight” response there is an activation of the limbic brain regions that gives way to neuroendocrine responses [1,10].

In this sense, FHA represents an adaptive response of the female organism to stress. More specifically, in the face of a greater expenditure of energy to ensure survival, the body begins to save on those functions that are not considered essential at that moment, such as reproduction. A first review that focused precisely on this aspect dates back to 1954 [11] and highlighted an increased incidence of amenorrhea during the First World War. Since the Second World War, amenorrhea began to be interpreted as a response of the body to emotional shock or as a “syndrome resulting from the combination of nutritional and psychic factors” [12].

More recently, it has been explained that individuals with FHA cope less well with stress in their autonomic responses and report significantly higher elevations of heart rate, systolic and diastolic blood pressure, and serum cortisol levels in comparison with controls [1,13,14].

At the psychophysiological level, the response to stress is characterized by an enhanced hypothalamic Corticotrophin-Releasing Hormone (CRH) release, which leads to the increase in Adreno-Cortico-Trophic Hormone (ACTH) secretion from the pituitary gland. At the end of the Hypothalamic–Pituitary–Adrenal (HPA) axis is the glucocorticoid release from the adrenal glands [1,10]. In individuals with FHA, the pulsatile release of GnRH is reduced. It is believed that the inhibition of the Hypothalamic–Pituitary–Gonadal (HPG) axis in women with FHA is caused by stress-related factors, including CRH [15]. This assumption is supported by a double-blind, randomized controlled trial by Pruneti et al. [13]. These authors, by administering Pivagabine (PVG), the drug of choice for the better modulation of the hypothalamic release of CRH, demonstrated that it is possible to modulate the adaptive response to stress and thus restore the menstrual cycle in women diagnosed with FHA. Furthermore, the strong involvement of the neuro-endocrine components was once again demonstrated.

### Aims

The goal of the present research is precisely to focus on psychophysiological stress as a key factor in FHA, excluding the conditions of physical suffering generated by low caloric intake and/or high energy consumption, which is why only women with normal body mass index were involved. The hypothesis that drove the study is that an alteration of the autonomic balance (the coordination between the two branches of the Autonomic Nervous System (ANS)–Parasympathetic (PANS) and Sympathetic (SANS) nervous systems) is present. To our knowledge, no studies have yet analyzed this component.

Lastly, the possible correlations between the psychophysiological values, obtained from the psychophysiological evaluation, and the different cognitive and behavioral variables, described through the psychopathological assessment, were investigated.

## 2. Materials and Methods

### 2.1. Participants and Study Design

In this case control study, fifty women aged between 14 and 26 years old were consecutively examined. The experimental group consisted of 25 patients (mean age = 21.1 ± 4.34) diagnosed with FHA. The control group was made up of 25 female students from the University of Parma (age-comparable, mean = 23.1 ± 3.27). The criteria for inclusion in the study were: normal BMI value (between 18.5–24.9); not on psychological/psychiatric or pharmacological treatment for psychological or medical problems; no medical or psychiatric diagnosis, except for the experimental group where women were included based on a confirmed diagnosis of FHA and a clinical condition characterized by the suspension of the menstrual cycle for at least three months (and regular previous menstrual cycles), LH value < 1, and no significant decrease in body weight in the past six months.

### 2.2. Procedures

A psychologist and a gynecologist recruited the participants while the patients were attending their first or second visit to the Institute of Clinical Obstetrics and Gynecology of the Hospital of Pisa (Italy). Data were collected at the Clinical Psychology Department of the Child Neuropsychiatric Centre at the St. Chiara University Hospital of Pisa (Italy). Patients were taken to a quiet room and were informed by a research assistant about the study procedures. After providing informed consent, patients completed the questionnaires and were familiarized with the equipment (e.g., cables) and the procedure for the recording of the psychophysiological parameters.

### 2.3. Measures

#### 2.3.1. Psychopathological Assessment

The Crown–Crisp Experiential Index (CCEI) [16,17] is a questionnaire composed of 48 items grouped into six scales that assess: Free Floating Anxiety (FFA), Phobic Anxiety (PHO), Depression (DEP) and Hysteroid Behavior (HYS), Obsessive traits (OBS), and Somatic complaints (SOM). Previous work suggests that the clinical cut-off set as ≥6 represents a clinically important threshold [18,19].

The Symptom Questionnaire—weekly version (SQ) [20,21] is a self-assessment questionnaire that investigates the state of well-being/distress perceived by the subject during a specific period. It contains four scales based on the factorial analysis of the psychological symptoms of Anxiety (A), Depression (D), Somatic Complaints (SC), and Hostility (H). This test evaluates both the presence of behavioral symptoms and cognitive aspects, such as the ability to relax, feel pleasure, and have a good disposition towards others. This test has weekly, daily, and hourly versions. The clinical cut-off corresponds to 4 for all the scales of the test.

The P Stress Questionnaire (PSQ) [22,23] is a tool made up of 32 items, grouped into six scales: Sense of Responsibility (SR), Vigor (V), Stress Disorders (SD), Precision and Punctuality (PP), Spare Time (ST), and Hyperactivity (H). It detects whether there is a present risk for stress-related physical disorders attributable to some characteristics of the personality configuration known as “Type A behavior”. Stanine scores have a distribution between 1 and 9, with a mean of 5 and a standard deviation of 1.96.

#### 2.3.2. Psychophysiological Assessment

A Psychophysiological Stress Profile (PSP) [24,25,26,27,28,29] structured in three phases was implemented. In the baseline phase (6 min), each patient was instructed to close their eyes and to remain still and relaxed. In the stress phase (4 min), a Mental Arithmetic Task (MAT) was presented to the participant. This task consists of subtracting the number 13 from the number 1007 and continuing to subtract 13 from each successive result that was obtained. Lastly, in the recovery phase (6 min) the patient was instructed to relax again. The following parameters were continuously registered: Surface Frontal Electromyography (sEMG)—where the electrical potential was detected by means two active electrodes placed 1 cm over the two eyebrows on the same line of the pupils and one reference electrode placed at the center of the front (2 cm of distance between poles); the Skin Conductance Level-Response (SCL-SCR)—where a very low intensity electrical direct current was attained by means two electrodes placed on the first and second finger of the non-dominant hand; Heart Rate (HR)—that consists of the detection of the electrical potential of cardiac muscle by the classic bipolar shunt for the electrocardiogram (ECG); and Peripheral Temperature (PT)—the peripheral body temperature was recorded by a thermistor with a device placed on the thenar eminence of the non-dominant hand. EMG and HR parameters were detected by means of surface disposable electrodes with 0.5 mm of active surface. For the SCR, two gold-plated electrodes were employed. For the PT, a very sensitive electronic thermometer (capable of evaluating fluctuations in temperature of less than 0.1°C) was utilized. The employed technology device was the “psycholab VD 13” by SATEM, Rome, Italy. The Modulab was connected by means an infrared cable with a PC and all the data were detected and processed by a PC soft VD 13SV VERSION 5.0 Works program software (by SATEM, Rome, Italy).

### 2.4. Statistical Analysis

Statistical analysis was performed using Microsoft Excel and IBM SPSS Statistics software (Version 28.0.1.0). The descriptive statistics of the scores obtained from the two samples in the CCEI, SQ, and PSQ were performed with the calculation of the mean (M) and standard deviation (SD). The average values of the parameters (sEMG, SCL-SCR, HR, and PT) of the PSP obtained by the two groups were also calculated.

To implement parametric statistical analyses, tests for Skewness, Kurtosis, and Kolmogorov–Smirnov were used in order to assess the normality of distribution.

The Independent Samples T-Test was used to evaluate the differences between the two groups (patients vs. control) in the comparison to the values obtained from the psychometric tests (CCEI, SQ, and PSQ) and psychophysiological parameters (sEMG, SCL-SCR, HR, and PT).

Lastly, a Pearson correlation was also performed using the same values.

## 3. Results

### 3.1. Description of the Sample

The socio-demographic characteristics of the sample are shown in Table 1.

### 3.2. Psychopathological Assessment

The statistical descriptive analyses conducted on the scores of the psychometric tests compiled by the group of patients showed that they exceed the clinical cut-off for most of the clinical sub-scales. Considering the psychological symptoms investigated with the CCEI, it was found that patients exceed the threshold of significance (≥6) for the sub-scales of anxiety (FFA), obsession (OBS), somatic complaints (SOM), depression (DEP), and hysteroid behavior (HYS). Still at a descriptive level, observing the scores obtained by the group of patients in the SQ questionnaire, scores well above the clinical cut-off (=4) emerge for all the symptoms (anxious activation (A), lowering of mood (D), somatic complaints (SC), and hostility/irritability (H)). No noteworthy data emerged from the questionnaire that investigates the presence of behaviors and lifestyles at risk for stress-related physical disorders (PSQ) because the group of patients exceeded the cut-off in the Sense of Responsibility (SR) sub-scale but a similar score was also obtained by the control group (Table 2).Furthermore, the calculation of the Independent Samples T-Test showed that the scores of the patients are significantly higher than the controls both in CCEI and SQ (Table 2).

### 3.3. Psychophysiological Assessment

With regard to the PSP, also in this case, the descriptive analyses demonstrated that the patients reported values that exceed the normal range. In particular, higher than normal psychophysiological values were recorded in the group of patients for muscle tension (sEMG), skin conductance (level and response, SCL/SCR), and peripheral temperature (PT) in all of the three recorded phases (baseline, stress, and recovery) (Table 3).

In addition, for all the psychophysiological parameters (sEMG, SCL-SCR, HR, PT), except for the HR in the stress phase, the difference between patients and control subjects is statistically significant (Table 4).

Lastly, a Pearson correlation analysis between the psychometric tests (CCEI, SQ, and PSQ) and the values of the stress phase of the PSP (sEMG, SCR, HR, PT) within the group of patients was conducted. The results highlight the considerable relationships between cognitive-behavioral aspects and emotional-psychophysiological components. More specifically, the values of muscle tension (sEMG) have a trend similar to the phobic anxiety scores (PHOB) of the CCEI and the tendency to adopt behaviors characterized by precision and punctuality (PP) investigated with the PSQ. Considering the HR parameter, a positive relationship emerges with obsessiveness (OBS) investigated through the CCEI and somatic complaints (SC) through the SQ. With regard to peripheral body temperature (PT), its augmentation corresponds to an increase in the depressive symptoms (D) of the SQ (Table 5).

## 4. Discussion

Performing a multidimensional assessment of patients with hypothalamic amenorrhea seems to be a very useful methodology. In fact, the results of the present study confirmed data from previous work in this area and allow for the addition of noteworthy aspects not investigated so far.

Considering the cognitive and behavioral components, the psychometric tests confirmed previous work regarding the presence of psychological distress in terms of anxious hyper activation and depression among women diagnosed with FHA [5,30,31]. Furthermore, other internalizing (somatic complaints and obsessions) and also externalizing (hostility and hysteric behavior) symptoms were found.

Because obsessive tendency has emerged, it confirms previous studies regarding mental rigidity and the use of control as a coping mechanism for the management of emotions [1,2]. This psychological aspect is considered an obstacle towards sexuality because it does not allow the woman to experience intimacy spontaneously and to “let go”. The tendency towards rigid perfectionism has also emerged in the precision and punctuality scale of the PSQ and the level of muscle tension measured with the PSP. Basically, mental rigidity favors the maintenance of high levels of psychophysiological alertness and does not allow the PANS (responsible for relaxation states and social and sexual activities) to prevail over the SANS.

Regarding the emotional-psychophysiological structure, the hypothesis that guided this research has been confirmed. In particular, observing the values of the psychophysiological parameters of the patients and comparing them with the women in the control group, a profile characterized by high levels of muscle tension, skin conductance, and heart rate and low levels of peripheral temperature emerges. This configuration is generally comparable to a condition characterized by autonomic hyper-arousal. More specifically, multiple characteristics of an excessively high level of sympathetic activity can be observed, namely higher than average values at baseline, marked reactivity to stimuli in the stress phase, and reduced emotional self-regulation skills necessary for post-stress psycho-physical recovery. These results show that in hypothalamic amenorrhea an alteration of the balance between the neuro-endocrine axes is also evident in terms of autonomic imbalance.

To better understand the connection between these two systems it is possible to refer to the first studies on stress, dating back to Selye [32]. This author was the first to describe stress in psychobiological terms with the General Adaptation Syndrome (GAS). According to his research, in the initial stage of any disease, there are general and nonspecific symptoms activated by the Central Nervous System (CNS) and the ANS. This univocal response to any adverse stimulus occurs in three phases. In the first phase (alarm), there is the activation of the SANS with the consequent activation of the medullary portion of the adrenal glands and secretion of adrenaline and noradrenaline. Both catecholamines increase cardiac output and, thus, increase the blood supply of skeletal muscles. The body mobilizes energy resources and directs them towards a fight or flight behavior. Furthermore, in this phase, the HPA axis is activated with secretion of glucocorticoids. In particular, cortisol, known as the “stress hormone”, triggers the conversion of proteins into glucose, involves lipids in the production of immediately available energy, increases blood flow, and activates behavioral responses. In the second phase (adaptation), the body tries to adapt to the new situation. Here, there is an overproduction of cortisol, and the body organizes itself to adapt and cope with the stimulus. Finally, in the third phase (exhaustion), there are two possible outcomes: the extinction of the stress response or a condition of functional exhaustion that occurs when the exposure to the stimulus continues for a long period and the organism does not have the resources to resist and/or to adapt further.

However, subjective experiences are never unique. It is well known in clinical psychology that stimuli endowed with the same stressful power does not cause the exact reaction in different individuals. The opposite is also true: stressful conditions of varying degrees can induce the same response in various people [25,26]. Precisely for this reason, it was decided to include some measures of psychological well-being in the multidimensional assessment. The results of the research showed a correspondence between symptoms of psychological distress and autonomic alteration. In particular, anxious traits, somatic complaints, and obsessiveness seem to favor a neurovegetative activation characterized by high muscle tension and increased heart rate. On the contrary, a lowering of mood corresponds to an increase in peripheral temperature. Confirming previous clinical studies [33,34], the various forms of depression would be characterized by a pathological condition of neurovegetative hypo activation where the autonomic imbalance leans towards the PANS. Therefore, although a trend towards psychophysiological hyper activation emerged from the total sample of patients, it is also possible that a subgroup among these women is experiencing something similar to an “emotional exhaustion”, a clinical condition characterized by neurovegetative hypo activation (with high peripheral temperature) and depressed mood, which is considered typical of chronic and prolonged stress [35,36].

The relationships that exist between the psychophysiological parameters and the psychological dimensions that characterize subjects with hypothalamic amenorrhea, testify that various aspects related to stress are involved, probably playing a fundamental role in its determination and maintenance. Some researchers consider the functional menstruation disorders as psychosomatic diseases for this reason [37].

Therefore, considering that FHA is a reflection of a response to significant endogenous stress, it is comprehensible that some authors [37,38,39] proposed the Cognitive-Behavioral Therapy (CBT) as a logical approach to lowering the stress levels. Promising results are described by Berga and colleagues [37] in 16 subjects with FHA randomized to CBT or observation for a 20 week period. The results were encouraging, with ovulation returning to six women in the CBT group and one woman in the observational group. Moreover, these data confirmed that endogenous stress is a major factor in the development and maintenance of FHA and that modification of stress response can restore normal menstrual cycle [38]. Although there are no studies in the literature regarding the effectiveness of couple therapy on FHA, it is possible to hypothesize that an intervention aimed at taking care of the relational aspect can also have positive effects. Previous research on sexual disorders of psychological origin has yielded promising results [40].

In closing, the results of our work confirm that the organism responds in accordance with two biological imperatives: the one of survival and the one of reproduction, the functions of which are supported by the complementary but antagonist activity of the SANS and PANS branches of the ANS. This perspective has been well described by Porges in his Polyvagal Theory (PVT) [41,42]. Functionally, when the environment is appraised as being safe, the defensive limbic structures are inhibited, enabling social engagement and calm visceral states to emerge. In contrast, some individuals experience a mismatch, and the nervous system appraises the environment as being dangerous. This mismatch results in physiological states that support fight, flight, or freeze behaviors, but not social engagement behaviors. In other words, if a person is in a fight-or-flight state, the social and sexual-reproductive system fails. More specifically, Porges highlights the role of the branch myelinated by the vagus nerve, which is mainly responsible for the functioning of the PANS (known as the ventral vagal complex) in social involvement.

According to a basic and simple perspective, such as the biological-evolutionary one, it is possible to understand that also sophisticated functions, such as sociality, relational behavior, and sexual activity, are still neurally represented in the most ancient systems of the brain and are almost partially under the control of the peripheral nervous system, the ANS.

### Limitations and Strengths

While the data provided by the present study are promising, confirmation through further research with larger sample sizes is needed. Therefore, larger patient groups would allow for more sophisticated statistical analyses. For instance, it might be interesting to evaluate the weight of a stressful event and psychological symptoms (including Anxiety, Depression, Somatic Complaints, and Hostility) in predicting and modulating the autonomic activation. Furthermore, it could also be useful to quantify the relationship between endocrine imbalance (in terms of Cortisol and LH levels) and autonomic activation (i.e., psychophysiological parameters).

However, the data provided by this research are promising because they accurately define the subjective experience of the suffering of women with FHA and the related autonomic imbalance. 

## 5. Conclusions

Psychological stress is frequently mentioned among the predisposing [28], precipitating, or perpetuating factors [42,43] of physical pathologies in most medical fields. Significant research is available in the literature aimed at identifying various stress response profiles, in which tests are used to elicit significant psycho-neuro-endocrine responses.

Considering that FHA is generally diagnosed through the exclusion of other possible diseases of gynecological and/or endocrinological relevance, our study aimed at investigating the psychophysiological stress that characterizes this medical condition. Excluding possible factors that could have caused FHA (i.e., alteration of body weight), women in whom psychological stress could have caused this condition were selected. In this research it was possible to more precisely define the condition of neurovegetative activation. Furthermore, this information was combined with psychological measures, capable of providing data with respect to the subjective experience of participants. This research is another example of how clinical psychology and clinical psychophysiology can provide a contribution through a multidisciplinary team that favors multidimensional assessment. 

Moreover, the increase in knowledge concerning the psychophysiological mechanisms connected to the stress response contributes to the development of new approaches to the treatment and management of the disease, including a pharmacological approach [43,44].

However, despite these reports, psychophysiological stress is not always described in its complexity (the endocrine, neurovegetative, and cognitive-behavioral aspects are equally important) and even more, clinical medicine units that benefit from the presence of psychology and counseling services are still few, as are the opportunities for multidisciplinary discussion between various health professionals to pursue the better management of disease and patient care.

## Figures and Tables

**Table 1:** Sociodemographic characteristics of participants.

	Patients Group (n = 25)	Control Group (n = 25)	Full Sample (n = 50)
Marital status, *N* (%)			
Single	6 (24%)	14 (56%)	20 (40%)
Married/partnered	18 (72%)	10 (40%)	28 (56%)
Divorced/widowed	-	1 (4%)	1 (4%)
Children ^a^, *N* (%)	1 (4%)	0 (0%)	1 (4%)
Highest educational level, *N* (%)			
Middle school	-	3 (12%)	3 (12%)
High school/some college	17 (68%)	18 (72%)	35 (70%)
University degree	8 (32%)	4 (16%)	12 (24%)
Employment, *N* (%)			
Unemployed	-	1 (4%)	1 (4%)
Student	23 (92%)	20 (80%)	43 (86%)
Employed	2 (8%)	4 (16%)	6 (12%)

Note. The ^a^ reflects the number and percentage of participants answering “yes” to this question.

**Table 2:** Average values and comparisons of the psychometric tests between patients and controls.

	Normal Range	Patients Group		Control Group		t	*p*
		M	SD	M	SD		
Crown-Crisp Experiential Index (CCEI)							
Free Floating Anxiety (FFA)	0–6	10.32	2.4	4.8	1.5	−2.45	<0.0001
Phobic Anxiety (PHO)	0–6	5.64	1.6	2.69	0.8	−2.27	<0.0001
Obsessive traits (OBS)	0–6	9.48	3.2	3.69	2.6	−1.68	<0.0005
Somatic Complaints (SOM)	0–6	7.28	1.8	2.91	0.6	−3.36	<0.0001
Depression (DEP)	0–6	9.8	3.3	2.69	1.8	−3.23	<0.0001
Hysteroid behavior (HYS)	0–6	8.52	2.7	3.17	0.4	−2.49	<0.0001
Symptom Questionnaire (SQ)							
Anxiety (A)	0–4	10.1	2.1	3.4	1,2	−3.83	<0.0001
Depression (D)	0–4	8.84	3.2	3	0.8	−2.72	<0.0001
Somatic Complaints (SC)	0–4	7.76	2.8	2.8	1.2	−1.15	<0.0005
Hostility (H)	0–4	7.76	3.1	2.5	2	−2.1	<0.0001
P Stress Questionnaire (PSQ)							
Sense of Responsibility (SR)	4–7	8.25	1.8	7.94	1.4	−0.2	n.s.
Vigor (V)	4–7	3.19	2	2.58	1.1	−0.32	n.s.
Stress Disorders (SD)	4–7	4.18	1.5	3.16	0.7	−0.93	n.s.
Precision and Punctuality (PP)	4–7	4.85	1.2	4.56	1.4	−0.16	n.s.
Spare Time (ST)	4–7	2.82	1.5	2.04	0.9	−0.41	n.s.
Hyperactivity (H)	4–7	5.47	2	5.22	1.9	−0.14	n.s.

**Table 3:** Average values obtained by the patients in the Psychophysiological Stress Profile (PSP).

	Normal Range	Baseline		Stress		Recovery	
		M	SD	M	SD	M	SD
surface Electromyography (sEMG, in µV)	1.7–2.5	4.65	1.7	5.22	1.7	4.85	1.6
Skin Conductance Level/Response (SCL/SCR, in µS)	2.2–6	9.6	6.3	12.71	7.4	12.63	7.7
Heart Rate (HR, in bpm)	60–90	77.13	9.7	79.15	13.6	77.11	10.3
Peripheral Temperature (PT, in °C)	31–33	27.5	2.6	27.6	2.4	27.6	2.6

**Table 4:** Comparison of the average values obtained by patients and controls in the Psychophysiological Stress Profile (PSP).

	Baseline		Stress		Recovery	
	t	*p*	t	*p*	t	*p*
surface Electromyography (sEMG, in µV)	−4.66	<0.001	−2.16	0.019	−4.39	<0.001
Skin Conductance Level/Response (SCL/SCR, in µS)	−4.9	<0.001	−5.66	<0.001	−5.41	<0.001
Heart Rate (HR, in bpm)	4.85	0.013	4.98	0.14	4.5	0.008
Peripheral Temperature (PT, in °C)	−2.31	<0.005	−1.07	<0.001	−2.51	<0.001

**Table 5:** Relationships between variables in the whole sample. Legend: ** *p* < 0.01. CCEI = Crown-Crisp Experiential Index; SQ = Symptom Questionnaire; PSQ = P Stress Questionnaire; PSP = Psychophysiological Stress Profile. Note: Only significant sub-scales were included in the table.

	1	2	3	4	5	6	7
1 CCEI Phobic Anxiety	1						
2 CCEI Obsessive traits	n.s.						
3 CCEI Depression	n.s.	n.s.					
4 SQ Somatic Complaints	n.s.	n.s.	n.s.				
5 PSQ Precision and Punctuality	n.s.	n.s.	n.s.	n.s.			
6 PSP surface Electromiography (µV)	0.44 **	n.s.	n.s.	n.s.	0.46 **		
7 PSP Heart Rate (bpm)	n.s.	0.44 **	n.s.	0.40 **	n.s.	n.s.	n.s.
8 PSP Peripheral Temperature (°C)	n.s.	n.s.	0.56 **	n.s.	n.s.	n.s.	n.s.

## Data Availability

The data presented in this study are available upon reasonable request from the corresponding author.

## References

[1] Gordon C.M., Ackerman K.E., Berga S.L., Kaplan J.R., Mastorakos G., Misra M., Murad M.H., Santoro N.F., Warren M.P. (2017). Functional Hypothalamic Amenorrhea: An Endocrine Society Clinical Practice Guideline. J. Clin. Endocrinol. Metab..

[2] Morrison A.E., Fleming S., Levy M.J. (2020). A review of the pathophysiology of functional hypothalamic amenorrhoea in women subject to psychological stress, disordered eating, excessive exercise or a combination of these factors. Clin. Endocrinol..

[3] Berga S.L., Mortola J.F., Girton L., Suh B., Laughlin G., Pham P., Yen S.S.C. (1989). Neuroendocrine Aberrations in Women With Functional Hypothalamic Amenorrhea*. J. Clin. Endocrinol. Metab..

[4] Dundon C.M., Rellini A.H., Tonani S., Santamaria V., Nappi R. (2010). Mood disorders and sexual functioning in women with functional hypothalamic amenorrhea. Fertil. Steril..

[5] Ryterska K., Kordek A., Załęska P. (2021). Has Menstruation Disappeared? Functional Hypothalamic Amenorrhea—What Is This Story about?. Nutrients.

[6] Gibson M.E.S., Fleming N., Zuijdwijk C., Dumont T. (2020). Where Have the Periods Gone? The Evaluation and Management of Functional Hypothalamic Amenorrhea. J. Clin. Res. Pediatr. Endocrinol..

[7] Sowińska-Przepiera E., Andrysiak-Mamos E., Jarząbek-Bielecka G., Walkowiak A., Osowicz-Korolonek L., Syrenicz M., Kędzia W., Syrenicz A. (2015). Czynnościowy podwzgórzowy brak miesiączki—trudności diagnostyczne, monitorowanie i leczenie. Endokrynol. Pol..

[8] Lania A., Gianotti L., Gagliardi I., Bondanelli M., Vena W., Ambrosio M.R. (2019). Functional hypothalamic and drug-induced amenorrhea: An overview. J. Endocrinol. Investig..

[9] Kyriakidis M., Caetano L., Anastasiadou N., Karasu T., Lashen H. (2016). Functional hypothalamic amenorrhoea: Leptin treatment, dietary intervention and counselling as alternatives to traditional practice—systematic review. Eur. J. Obstet. Gynecol. Reprod. Biol..

[10] McCosh R.B., Breen K.M., Kauffman A.S. (2019). Neural and endocrine mechanisms underlying stress-induced suppression of pulsatile LH secretion. Mol. Cell. Endocrinol..

[11] Daniels G.E., Poe J., Easser R., Monroe R., Kelley K. (1954). Psychological Correlations with Secondary Amenorrhea. Psychosom. Med..

[12] Copelman L.S. (1948). L′aménorrhée des déportées, syndrome neuro-ergonal [The amenorrhea of the deportees, neuro-ergonal syndrome]. Rev. Pathol. Comp..

[13] Pruneti C.A., Petraglia F., Rossi F., Rota S., Stomati M., Luisi M., Genazzani M.R. (1999). Studio, in doppio cieco, degli effetti a livello comportamentale, psicofisiologico ed endocrinologico in pazienti con amenorrea ipotalamica, di un trattamento farmacologico a breve termine con Pivagabina (PVG). G. Ital. Di Psicopatol..

[14] Gallinelli A., Matteo M.L., Volpe A., Facchinetti F. (2000). Autonomic and neuroendocrine responses to stress in patients with functional hypothalamic secondary amenorrhea. Fertil. Steril..

[15] Barbarino A., De Marinis L., Tofani A., della Casa S., D’Amico C., Mancini A., Corsello S.M., Sciuto R., Barini A. (1989). Corticotropin-Releasing Hormone Inhibition of Gonadotropin Release and the Effect of Opioid Blockade. J. Clin. Endocrinol. Metab..

[16] Crown S., Crisp A.H. (1966). A Short Clinical Diagnostic Self-rating Scale for Psychoneurotic Patients. Br. J. Psychiatry.

[17] Birtchnell J., Evans C., Kennard J. (1988). The total score of the Crown-Crisp Experiential Index: A useful and valid measure of psychoneurotic pathology. Psychol. Psychother. Theory Res. Pr..

[18] McGrath M., Kawachi I., Ascherio A., Colditz G.A., Hunter D.J., De Vivo I. (2004). Association Between Catechol-*O*-Methyltransferase and Phobic Anxiety. Am. J. Psychiatry.

[19] Okereke O.I., Prescott J., Wong J.Y.Y., Han J., Rexrode K.M., De Vivo I. (2012). High Phobic Anxiety Is Related to Lower Leukocyte Telomere Length in Women. PLoS ONE.

[20] Kellner R. (1976). Abridged manual of the Symptom Questionnaire.

[21] Fava G.A., Kellner R., Perini G.I., Fava M., Michelacci L., Munari F., Evangelisti L.P., Grandi S., Bernardi M., Mastrogiacomo I. (1983). Italian Validation of the Symptom Rating Test (SRT) and Symptom Questionnaire (SQ)^*^. Can. J. Psychiatry.

[22] Pruneti C.A., Mazzei M.G., L’abbate A., Baracchini-Muratorio G. (1988). Aspetti psicopatologici nel paziente cardiovascolare: Possibile ruolo prognostico di alcune caratteristiche della personalità. Ann. Di Neurol. E Psichiatr..

[23] Pruneti C. (2011). The P Stress Questionnaire: A new tool for the evaluation of stress- related behaviors. Eur. J. Clin. Psychol. Psychiatry.

[24] Fuller G.D. (1979). Biofeedback Methods and Procedures in Clinical Practice.

[25] Pruneti C., Lento R.M., Fante C., Carrozzo E., Fontana F. (2010). Autonomic arousal and differential diagnosis in clinical psychology and psychopathology. J. Psychopathol..

[26] Pruneti C., Fontana F., Carrozzo E., Fante C. (2011). Autonomic Reactivity, Emotions and Stress Response in Psychopathology. Appl. Psychophysiol. Biofeedback.

[27] Pruneti C., Vanello N., Paterni M., Landini L., Guidotti S., Ferdeghini E.M. (2021). Combined functional magnetic resonance imaging and skin conductance to detect localized neural response to psychological stress: A pilot study. Arch. Ital. De Biol..

[28] Cosentino C., Sgromo D., Merisio C., Berretta R., Pruneti C. (2018). Psychophysiological Adjustment to Ovarian Cancer: Preliminary Study on Italian Women Condition. Appl. Psychophysiol. Biofeedback.

[29] De Vincenzo F., Cosentino C., Quinto R.M., Di Leo S., Contardi A., Guidotti S., Iani L., Pruneti C. (2022). Psychological adjustment and heart rate variability in ovarian cancer survivors. Mediterr. J. Clin. Psychol..

[30] Pauli S.A., Berga S.L. (2010). Athletic amenorrhea: Energy deficit or psychogenic challenge?. Ann. N. Y. Acad. Sci..

[31] Bomba M., Corbetta F., Bonini L., Gambera A., Tremolizzo L., Neri F., Nacinovich R. (2013). Psychopathological traits of adolescents with functional hypothalamic amenorrhea: A comparison with anorexia nervosa. Eat. Weight Disord. Stud. Anorex. Bulim. Obes..

[32] Selye H. (1998). A Syndrome Produced by Diverse Nocuous Agents. J. Neuropsychiatry Clin. Neurosci..

[33] Kim A.Y., Jang E.H., Choi K.W., Jeon H.J., Byun S., Sim J.Y., Choi J.H., Yu H.Y. (2019). Skin conductance responses in Major Depressive Disorder (MDD) under mental arithmetic stress. PLoS ONE.

[34] Schiweck C., Piette D., Berckmans D., Claes S., Vrieze E. (2019). Heart rate and high frequency heart rate variability during stress as biomarker for clinical depression. A systematic review. Psychol. Med..

[35] Pruneti C., Cosentino C., Sgromo M., Innocenti A. (2014). Skin Conductance Response as a decisive variable in individuals with a DSM-IV TR Axis I diagnosis. JMED Res..

[36] Pruneti C., Saccò M., Cosentino C., Sgromo D. (2016). Relevance of Autonomic Arousal in the Stress Response in Psychopathology. J. Basic Appl. Sci..

[37] Berga S.L., Marcus M.D., Loucks T.L., Hlastala S., Ringham R., A Krohn M. (2003). Recovery of ovarian activity in women with functional hypothalamic amenorrhea who were treated with cognitive behavior therapy. Fertil. Steril..

[38] Liu J.H., Bill A.H. (2008). Stress-Associated or Functional Hypothalamic Amenorrhea in the Adolescent. Ann. N. Y. Acad. Sci..

[39] Michopoulos V., Mancini F., Loucks T.L., Berga S.L. (2013). Neuroendocrine recovery initiated by cognitive behavioral therapy in women with functional hypothalamic amenorrhea: A randomized, controlled trial. Fertil. Steril..

[40] Starc A., Trampuš M., Jukić D.P., Rotim C., Jukić T., Mivšek A.P., Grgas-Bile C. (2019). Infertility and Sexual Dysfunctions: A Systematic Literature Review. Acta Clin. Croat..

[41] Porges S.W. (2009). The polyvagal theory: New insights into adaptive reactions of the autonomic nervous system. Clevel. Clin. J. Med..

[42] Porges S.W. (2021). Polyvagal Theory: A biobehavioral journey to sociality. Compr. Psychoneuroendocrinol..

[43] Bonaguidi F., Michelassi C., Trivella M.G., Carpeggiani C., Pruneti C.A., Cesana G., L′Abbate A. (1996). Cattell′s 16 PF and PSY Inventory: Relationship between Personality Traits and Behavioral Responses in Patients with Acute Myocardial Infarction. Psychol. Rep..

[44] Pruneti C., Giusti M., Boem A., Luisi M. (2002). Behavioral, psycho-physiological and salivary cortisol modifications after short-term alprazolam treatment in patients with recent myocardial infarction. Ital. Heart J..

